# β-Carotene Production from *Dunaliella salina* Cultivated with Bicarbonate as Carbon Source

**DOI:** 10.4014/jmb.1910.10035

**Published:** 2020-03-13

**Authors:** Yimei Xi, Jinghan Wang, Song Xue, Zhanyou Chi

**Affiliations:** School of Bioengineering, Dalian University of Technology, Dalian 116024, P.R. China

**Keywords:** *Dunaliella salina*, β-carotene, bicarbonate, microelement, carbon source

## Abstract

Bicarbonate has been considered as a better approach for supplying CO_2_ to microalgae cells microenvironments than gas bubbling owing to cost-effectiveness and easy operation. However, the β-carotene production was too low in *Dunaliella salina* cultivated with bicarbonate in previous studies. Also, the difference in photosynthetic efficiency between these two carbon sources (bicarbonate and CO_2_) has seldom been discussed. In this study, the culture conditions, including NaHCO_3_, Ca^2+^, Mg^2+^ and microelement concentrations, were optimized when bicarbonate was used as carbon source. Under optimized condition, a maximum biomass concentration of 0.71 g/l^-1^ and corresponding β-carotene content of 4.76% were obtained, with β-carotene yield of 32.0 mg/l^-1^, much higher than previous studies with NaHCO_3_. Finally, these optimized conditions with bicarbonate were compared with CO_2_ bubbling by online monitoring. There was a notable difference in *F_v_/F_m_* value between cultivations with bicarbonate and CO_2_, but there was no difference in the *F_v_/F_m_* periodic changing patterns. This indicates that the high concentration of NaHCO_3_ used in this study served as a stress factor for β- carotene accumulation, although high productivity of biomass was still obtained.

## Introduction

*Dunaliella salina* is a halo-tolerant microalgae species that accumulates high levels of β-carotene [1, 2]. Although it has been used in commercial production of carotene for several decades, its production cost is still very high, limiting its application to only high-value nutraceuticals [3]. For products of lower value but much larger market size, such as animal and aquaculture feed additives, the current production cost of *Dunaliella* is much too high to be economically feasible, and needs to be significantly reduced.

Carbon accounts for about 50% of microalgae’s dry weight, and its supply is very important in the development of cost-effective microalgae cultivation processes. In conventional cultivation systems, inorganic carbon is usually supplied as gaseous CO_2_, which has caused serious technological difficulties in photobioreactor (PBR) design and operation [4,5,6], ultimately resulting in high production cost. Recently, bicarbonate has been suggested as a better approach to supplying carbon, with the advantages of easy transportation, handling, and storage. In such conditions, an aeration-free PBR can be used as a low-cost cultivation system [7-10].

There have been some reports on employing sodium bicarbonate to stimulate β-carotene accumulation in *D. salina* [8, 9, 11, 12]. However, β-carotene production reached in these studies was much lower than the conventional cultivation with CO_2_, amounting to only 8.25 ± 0.01 mg/l [12], 7.10 ± 0.08 mg/l [9], and 20.43 ± 2.84 mg/l [8], respectively. These levels are too low to be used as routine practice in large-scale production. As a matter of fact, when bicarbonate is used as carbon source, the optimal culture condition for β-carotene accumulation may be different from that with CO_2_. In this situation, it is necessary to disclose the effects of different factors on cell growth and β-carotene accumulation with bicarbonate as carbon source.

Also, when bicarbonate is used in the culture, pH drift is inevitable, and HCO_3_^-^ converts to CO_3_^2-^ at high pH. Seawater is usually used as culture medium for *Dunaliella* sp., and the high concentration of Ca^2+^ and Mg^2+^ in it may precipitate with CO_3_^2-^, since CaCO_3_ precipitation usually occurs when pH exceeds 8.5, and Mg(OH)_2_ precipitation forms when pH exceeds 10. Although pH adjustment with acid can avoid this precipitation, it is not practical for outdoor large-scale cultivation since additional chemicals and sophisticated equipment are required. An alternative method to avoid this precipitation is reducing the concentrations of Ca^2+^ and Mg^2+^, but this reduction to a certain level may limit the production of carotene. Thus, the effects of Ca^2+^ and Mg^2+^ concentrations need to be carefully investigated.

β-carotene accumulation is usually triggered by environmental stress such as light, temperature, salinity, or nitrogen depletion [13, 14]. *F_v_/F_m_* is the photosystem II (PS II) maximum photochemical quantum yield. It is sensitive to stress conditions for microalgae, and has been established as a quantitative indicator that shows certain stress levels. For example, the value of *F_v_/F_m_* can reflect fatty acid accumulation in response to nitrogen depletion [15]. *F_v_/F_m_* was also used as an indicator in *Dunaliella* sp. in previous study to disclose the mechanism of β-carotene accumulation in response to different stresses [12]. Thus, the *F_v_/F_m_* was monitored in this study, to compare the stress the cells experienced in cultivation with either bicarbonate or CO_2_, which finally affects the β-carotene accumulation.

The present study, therefore, investigated the effects of various factors on *D. salina* growth and β-carotene production when sodium bicarbonate is used as carbon source. Different concentrations of sodium bicarbonate, as well as Ca^2+^ and Mg^2+^ were investigated for *D. salina* growth and β-carotene accumulation. Central composite design experiments were carried out for studying the effect of microelements on biomass and β-carotene content of *D. salina*. Finally, maximal PS II quantum yield and β-carotene content were analyzed and compared between bicarbonate and CO_2_-based cultivation.

## Materials and Methods

### Strain and Medium

The microalgae *D. salina* CCAP 19/18 was purchased from Culture Collection of Algae and Protozoa agencies (UK), and it was maintained in Artificial Sea Water (ASW). The nutrient medium was 1.5 M NaCl, 5 mM KNO_3_, 4.5 mM MgCl_2_·6H_2_O, 0.5 mM MgSO_4_·7H_2_O, 3 mM CaCl_2_·2H_2_O, 0.13 mM K_2_HPO_4_, 0.02 mM FeCl_3_, 0.02 mM EDTA, 25 mM NaHCO_3_, 1 ml of trace elements stock with 50 mM H_3_BO_3_, 10 mM MnCl_2_·4H_2_O, 0.8 mM ZnSO_4_·7H_2_O, 1.0 mM CuSO_4_·5H_2_O, 2 mM NaMoO_4_·2H_2_O, 1.5 mM NaVO_3_, 0.8 mM CoCl_2_ ·6H_2_O, and the pH was adjusted to 7.5 by addition 40mM of Tris-buffer [16]. Before inoculation, microalgae were cultivated in batch mode to promote fast growth in 500 mL conical flasks with light intensity of 40 μmol/m^-2^/s^-1^ and alternating 12 h/ 12 h light/dark cycles. After inoculation, the initial cell concentration in each horizontal photobioreactor (PBR) was about 0.2 ×10^6^ cells/ml^-1^. The horizontal PBRs were polystyrene boxes of 12 cm × 12 cm, with a working volume of 250 ml and a light path of 20 mm. Light was provided by white LEDs, with intensity on the top surface of the PBR controlled at 200 μmol/m^-2^/s^-1^ and under 12 h/12 h light/dark cycles. Cultivation temperature was controlled at 25 ± 0.5°C in the illumination incubator. Each experiment below was carried out in triplicate.

### Growth Analysis

Cell numbers were counted daily using a hematocyto-meter. Dry weight (DW, g/l) was measured by using pre-weighed Whatman GF/C filters [17, 18]. Ten milliliter cultures were filtered and washed three times with 2 ml 0.5 M ammonium bicarbonate and then were dried below 60°C for over 16 h until the weight was constant. The DW of the microalgae cells was calculated according to the final weight and volume of the filtered sample.

Analysis of microalgal β-carotene was based on methods described in Mojaat [19]. For β-carotene content measurement, 10 mg of dried biomass was extracted with 1 ml acetone and vortexed for 20 s. Then, it was centrifuged at 10,000 ×g for 10 min. This extraction was repeated twice. The extracts were filtered by 0.45-μm pore size (PTFE) membrane syringe filters (1.7 cm^2^). All extracts were treated using amber glass vials with screw caps to protect carotenoids from degradation under light. The β-carotene analysis was carried out by High-Performance Liquid Chromatography (HPLC, Agilent Technologies 1100, USA). The mobile phase was 10% acetonitrile and 90% methanol. The flow rate was 1 ml/min^-1^, and the detection wavelength was 452 nm. The standard sample of β-carotene was purchased from Sigma (Sigma-Aldrich, USA).

### Experimental Design and Data Analysis

**Influence of sodium bicarbonate concentrations on *D. salina* growth.** To test the effect of NaHCO_3_ concentrations on *D. salina* CCAP 19/18, six concentration gradients were selected, *i.e.*, 25, 50, 100, 200, 300, and 500 mM. The concentrations of NaCl, Ca^2+^, and Mg^2+^ in the culture medium were 1.5 M, 3.0 mM, and 5.0 mM, respectively.

**Influence of Ca^2+^ and Mg^2+^concentrations on *D. salina* growth.** When optimizing the concentrations of Ca^2+^ and Mg^2+^ for *D. salina* growth, gradients of Ca^2+^ and Mg^2+^ concentrations were from 0.3 to 3.0 mM, and from 0.5 to 5.0 mM, respectively, as listed in Table 1. Optimized NaHCO_3_ concentration (200 mM) was adopted for all cultures with various Ca^2+^ and Mg^2+^ concentrations.

### Central Composite Design

With a Plackett-Burman (PB) design, three significant microelements listed in Table 2 were screened from FeCl_3_·6H_2_O, H_3_BO_3_, ZnSO_4_·7H_2_O, CoCl_2_·6H_2_O, CuSO_4_·5H_2_O, MnCl_2_·4H_2_O, NaMoO_4_·2H_2_O, and NaVO_3_, as displayed in Table S1. A central composite design was used to investigate their effects on dry cell weight and *D. salina* β-carotene yield. The design matrix was a 2^4^ full factor design combined with five central points, and eight axial points where one variable was set at an extreme level while other variables were set at their central point levels. The coded and real values of each parameter are shown in Table 2. Based on Table 3, the responses (cell dry weight and β-carotene yield) were correlated as a function of variables by a second-order polynomial equation, *i.e.*,

Y = β0 ΣβiXi++ΣβiXi2 Σ+βijXiXj (1)

where *Y* is the predicted response, *β* is the coefficient of the equation, and *x*_i_ and *x*_j_ are the coded levels of variables *i* and *j*, respectively. The software Design-Expert (Stat-Ease Inc., USA) was adopted for this correlation through non-linear regression. An *F*-test was used to evaluate the significance of the models.

### NaHCO_3_ vs. CO_2_ Cultivation with Online Monitoring

To confirm the experimental results obtained from the NaHCO_3_, *D. salina* was cultivated with a multi-device-equipped flat-plate photobioreactor (PBR)-Algal station [15] in both NaHCO_3_-based (optimal NaHCO_3_ concentration) and CO_2_-based (2%) cultivations. Light was provided by white LEDs, with intensity on the top surface of the PBR controlled at 200 μmol/m^-2^/s^-1^ and under 12 h/12 h light/dark cycles. Light path of the PBR was 20 mm and total cultivation volume was 1 L. The cultivation temperature was automatically controlled at 25±0.5°C. Cultures in the PBR were agitated with 0.2-μm membrane-filtered air at 200 ml/min (NaHCO_3_-based culture) or with 2% CO_2_ (CO_2_-based culture). Maximal PS II quantum yield (*F_v_/F_m_*), OD, and pH value were recorded online.

## Results

### Effect of NaHCO_3_ Concentration on Cell Growth and β-Carotene Accumulation

**Fig. 1:**
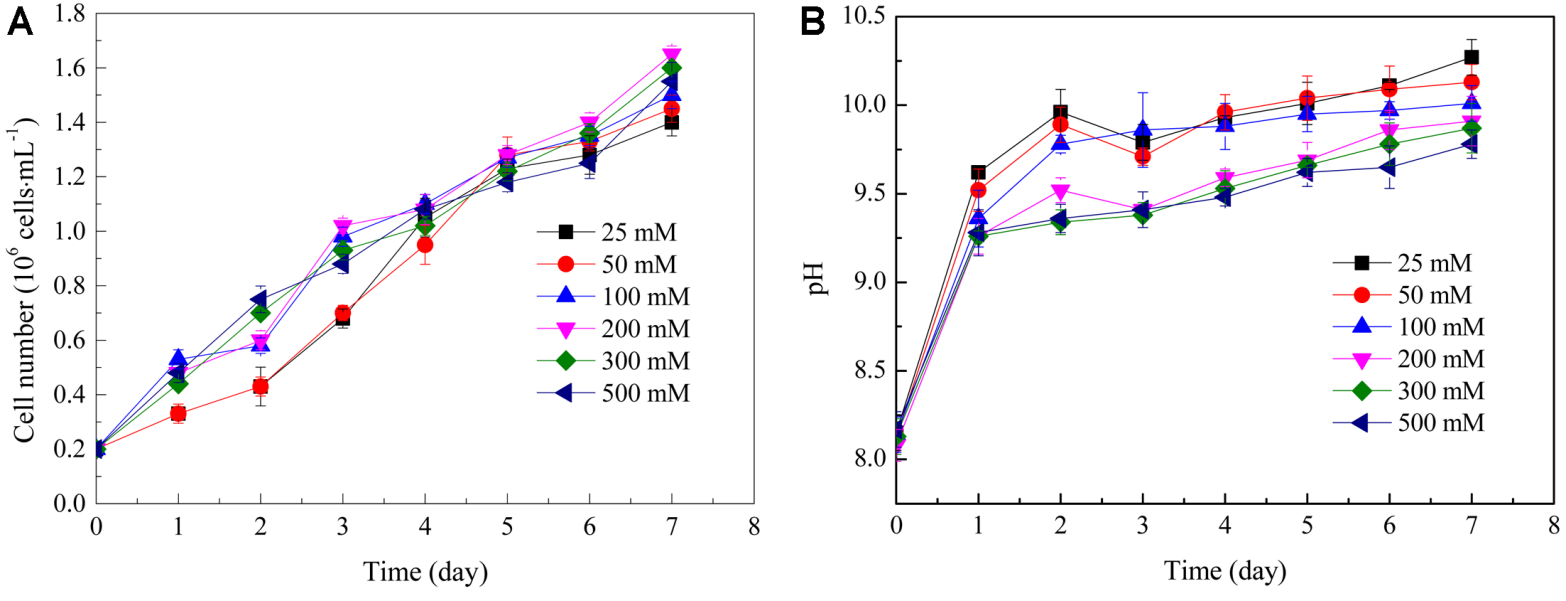
Effect of different concentrations of NaHCO_3_ on (A) *D. salina* cell density; (B) pH of the culturing broth. Values represented as mean ± SD (*n* = 3).

**Fig. 2:**
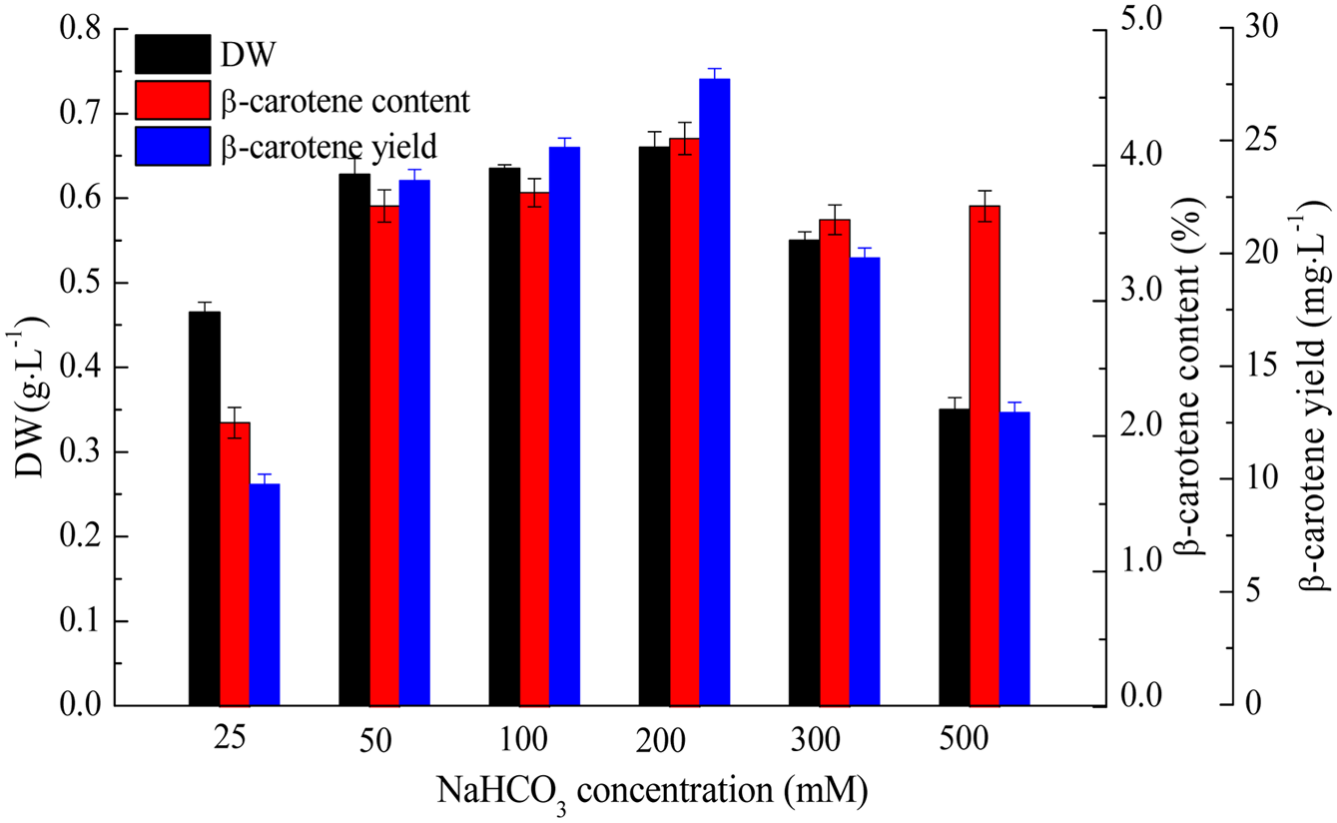
Effect of NaHCO_3_ concentrations on DW, β-carotene content, and β-carotene yield of *D. salina*. Values represented as mean ± SD (*n* = 3).

Growth curves for *D. salina* cultivated under a series of NaHCO_3_ concentrations are shown in Fig.1A, indicating *D. salina* could grow well in medium with NaHCO_3_ as carbon source. Cell densities of 25, 50, and 100 mM NaHCO_3_ cultures increased rapidly during the first 3 days of cultivation, and were higher than those of 200, 300, and 500 mM cultures. After that, cell density decreased in cultures with NaHCO_3_ concentrations lower than 100 mM. The lowest cell density was observed in 25 mM NaHCO_3_ and the highest cell density was recorded in 200 mM NaHCO_3_ culture, which was 1.65 × 10^6^ cells/ml^-1^ on Day 7. As far as the DW is concerned, the highest DW was obtained on Day 7 with 200 mM NaHCO_3_ concentration (0.66 ± 0.02 g/l), whereas the lowest DW of 0.35 ± 0.01 g/l was obtained in culture with 50 mM NaHCO_3_ (Fig. 2). It was interesting that *D. salina* was able to grow in culture with 500 mM NaHCO_3_, although 200 mM NaHCO_3_ enabled the fastest growth.

Correspondingly, pH variations of these cultures were displayed in Fig. 1B. The pH values of the six cultures spiked on Day 1 then increased slowly afterwards. At Day 7, the highest and lowest pH values were obtained in 25 mM culture (pH = 10.3) and 500 mM culture (pH = 9.8), respectively. For cultures with ≥100 mM NaHCO_3_, pHs in these cultures were below 10.0 during the whole cultivation time. When correlating pH with microalgal growth, cultures with higher pH resulted in lower growth rates, indicating that elevated pH inhibited growth of *D. salina*, although it was able to grow at pH value higher than 10.0. Moreover, these results indicated that NaHCO_3_ had a strong buffering effect on pH.

When correlating NaHCO_3_ concentration with DW, the cellular β-carotene content levels of *D. salina* grown under different NaHCO_3_ concentrations were displayed in Fig. 2. The highest β-carotene content of 4.20 ± 0.12% DW was obtained in culture with 200 mM NaHCO_3_, and the corresponding β-carotene yield was 27.72 ± 1.65 mg/l. In contrast, the lowest β-carotene content of 2.1 ± 0.11% was obtained in 25 mM culture, reaching β-carotene yield of 9.77±0.45mg/l. In culture with 500mM NaHCO_3_ concentration, although high β-carotene content of 3.7 ± 0.11% was obtained, the overall available β-carotene yield was only 12.95 ± 0.46 mg/l owing to limited microalgal growth. From these results, it was implied that NaHCO_3_ concentration could greatly influence cell growth and β-carotene accumulation of *D. salina*, and 200 mM was optimal for both biomass and β-carotene production in this study.

### Effect of Ca^2+^ and Mg^2+^ Concentrations on Cell Growth and β-Carotene Accumulation

**Fig. 3:**
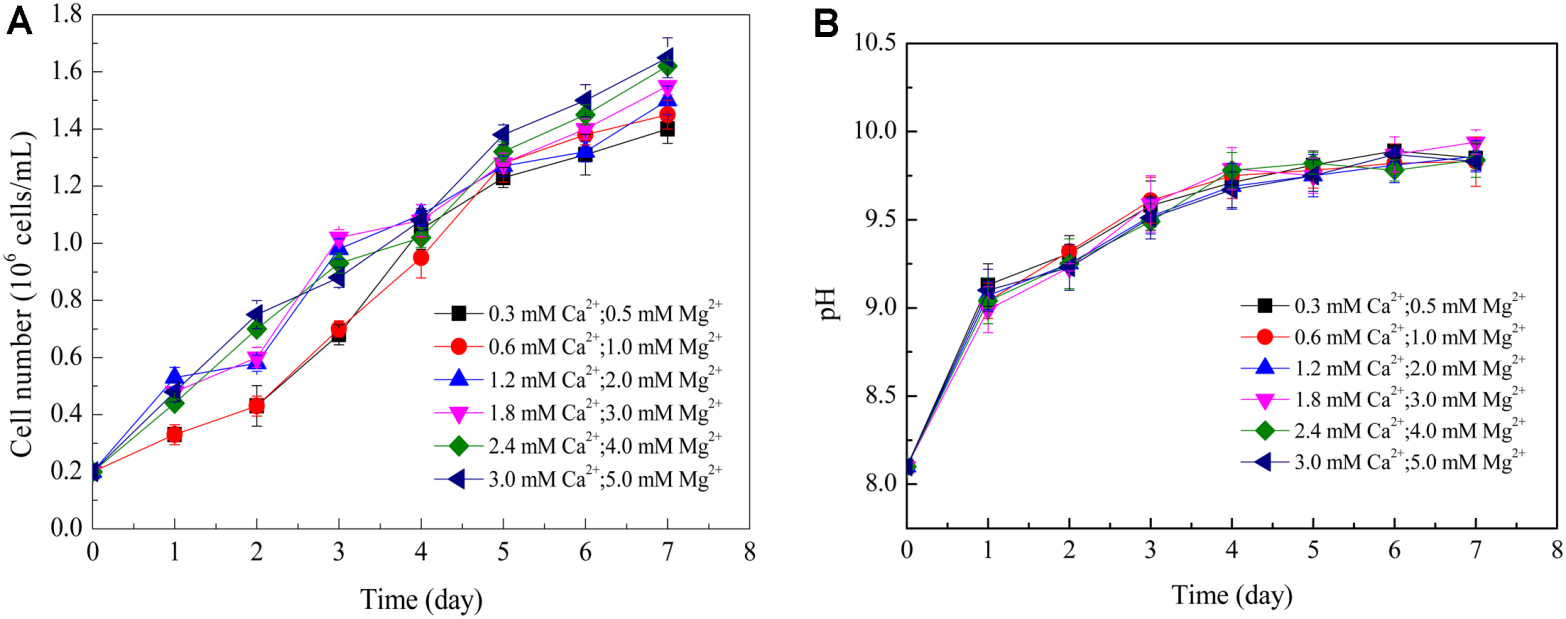
Effect of Ca^2+^ and Mg^2+^ concentrations on (A) cell number of *D. salina*; (B) pH of the culturing broth. Values represented as mean ± SD (*n* = 3).

The correlation between Ca^2+^, Mg^2+^ and growth of *D. salina* was shown in Fig. 3A. Cell numbers ranged from 1.40 to 1.65 × 10^6^ cells/ml under different Ca^2+^ and Mg^2+^ concentrations, and significant differences were observed among all cultures on Day 7 (*p* = 0.03 < 0.1). The highest cell number was obtained in culture with 3.0 mM Ca^2+^ and 5.0 mM Mg^2+^, while the lowest cell number was obtained in culture with 0.3 mM Ca^2+^ and 0.5 mM Mg^2+^. pH values of all cultures on Day 7 displayed insignificant differences (*p* = 0.12 > 0.1), with values around 9.8 (Fig. 3B).

**Fig. 4:**
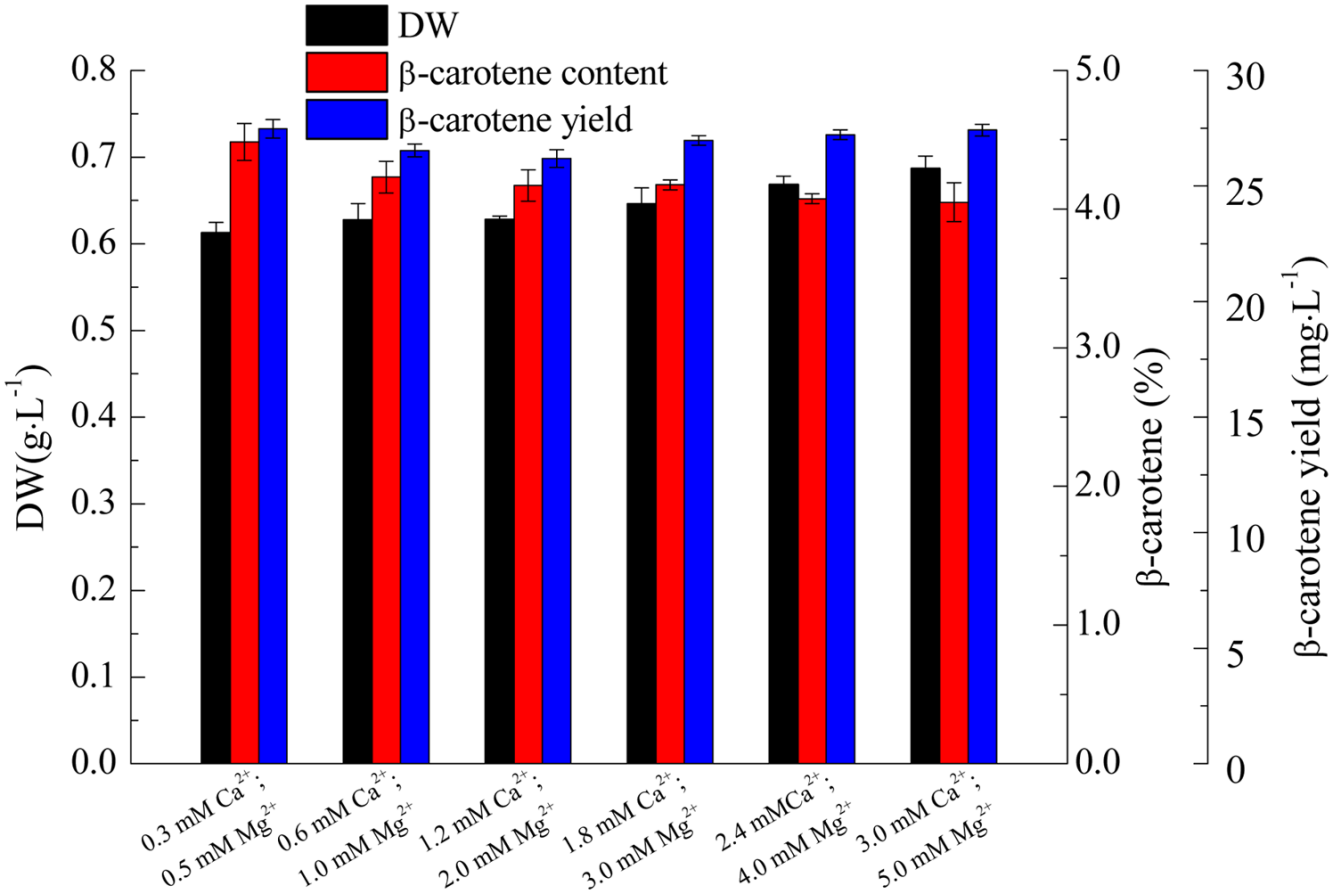
Effect of Ca^2+^, Mg^2+^ concentrations on DW and β-carotene accumulation of *D. salina*. Values represent as mean ± SD (*n* = 3).

As shown in Fig. 4, DW was positively correlated with Ca2 + and Mg2 + concentrations, with the highest and lowest DW (0.69 g/l and 0.61 g/l) respectively at the same culturing conditions regarding cell numbers (Fig. 3A). In contrast, β-carotene content was negatively correlated with Ca2 + and Mg2 + concentrations, with the highest and lowest β-carotene content (respectively 4.5% and 4.1%) obtained at culturing conditions opposite to those of cell density and DW. It was noteworthy that β-carotene yield obtained in cultures with different Ca^2+^ and Mg2 + were around the same levels (26.2 to 27.5 mg/l), displaying no significant differences (*p* = 0.87 > 0.10). Thus, low concentration Ca^2+^ and Mg^2+^ of 0.3 mM and 0.5 mM respectively, are adequate for β-carotene accumulation.

### Effect of Micronutrients on Cell Growth and β-Carotene Accumulation

**Fig. 5:**
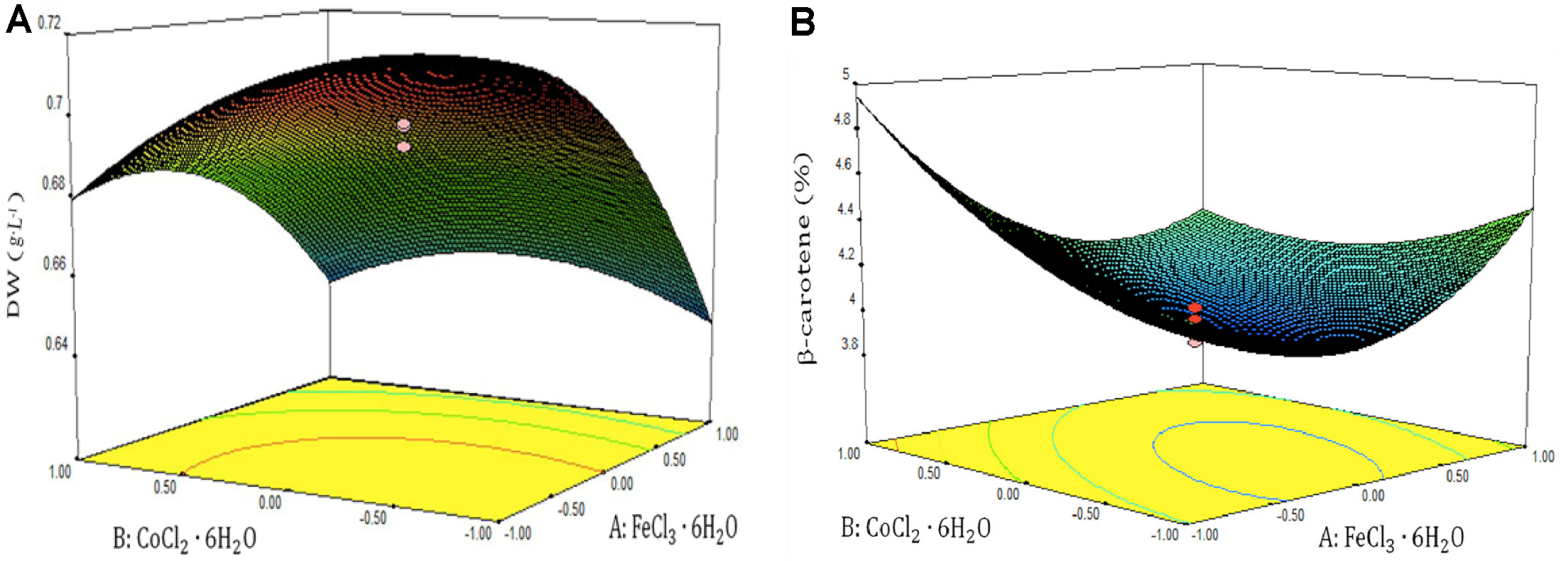
Three-dimensional response surface plot of (A) DW and (B) β-carotene content as a function of FeCl_3_·6H_2_O and CoCl_2_·6H_2_O.

**Optimization of significant factors.** For central composite design, the central point values and level ranges of three significant factors were selected according to the PB design results (Table S1). As shown in Table 3, the central point in the central composite design was repeated five times, and standard deviation of these five replicates used to determine the experimental errors were 0.008 g/l for DW, and 0.07% for β-carotene content. The experimental data of DW and β-carotene content in Table 3 were correlated as functions of the three variables by a second-order polynomial equation using the Design-Expert software. The coefficient values in Eq. (1) and their *p*-values and *F*-values were listed in Table 4. The optimal value of the three variables were derived by Design-Expert software, and the maximum dry cell weight of 0.71 g/l was obtained in culture with 1.85 μM FeCl_3_·6H_2_O, 1.6 μM CoCl_2_·6H_2_O, and 1.48 μM NaVO_3_ concentrations. The maximum β-carotene content of 4.76% was obtained in culture with 5.92 μM FeCl_3_·6H_2_O, 2.23 μM CoCl_2_·6H_2_O, and 2.05 μM NaVO_3_. From *p*-levels in Table 4 and the response surfaces in Fig. 5, it is evident that microelements, especially Fe*3+* and Co^2+^ and their concentrations, can have significant influences on *D. salina* growth and β-carotene accumulation.

Three-dimension surface responses were plotted to illustrate the relationship between the variables and their responses. Because the statistical analysis indicated that FeCl_3_·6H_2_O and CoCl_2_·6H_2_O concentration had more significant effects on the responses than NaVO_3_ concentration (*p*-level, Table 4), the responses (DW and β-carotene content) were plotted as the functions of FeCl_3_·6H_2_O and CoCl_2_·6H_2_O. As shown in Fig. 5A, low concentrations of FeCl_3_·6H_2_O and CoCl_2_·6H_2_O led to an increase in DW, but β-carotene content increased with augmentation of CoCl_2_·6H_2_O and FeCl_3_·6H_2_O concentrations (Fig. 5B).

### Verification of Optimized Culture Conditions

The optimal conditions determined from the central composite design were verified by comparing the experimental data obtained at these conditions with that predicted from central composite design (Eq. (1) and Table 4). Three verification experiments were conducted, which were respectively optimal for DW, β-carotene content, and both DW and β-carotene content (Table 5). For DW experiment under optimized condition, the experimental data was 0.77 ± 0.01 g/l, while the predicted value was 0.86 g/l, indicating 9–10% deviation. As for the condition optimal for β-carotene content, experimental data was 4.78% ± 0.04, while the predicted value was 4.73%, indicating a deviation of less than 5% (Table 5). With the second-order polynomial equation obtained in this study, the calculated DW (before optimizing the microelements) was 0.67 g/l, and the β-carotene content was 4.2%. After the microelement optimization, DW was calculated as 0.78 g/l, and β-carotene content was 4.78%(Table 5). These experiments verified the effectiveness of the model developed in this study.

### Comparisons between Cultivations with NaHCO_3_ and CO_2_

**Fig. 6:**
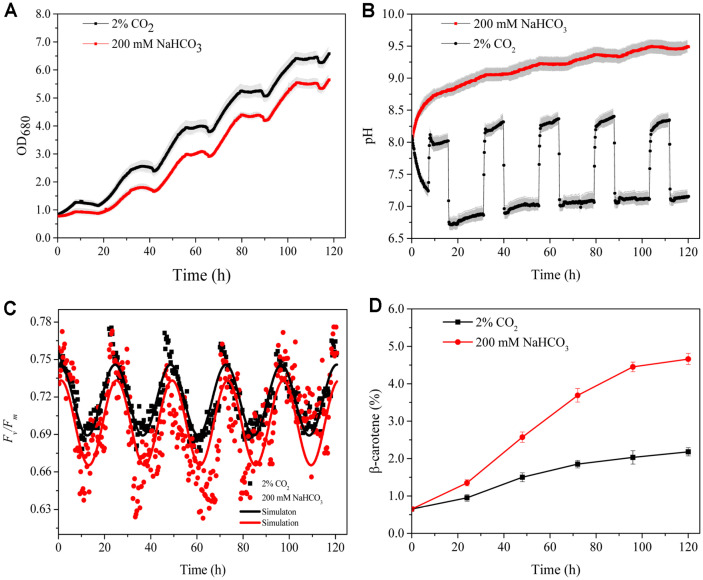
NaHCO_3_-based cultivation vs. CO_2_-based cultivation by online monitoring (A) Growth curve of *D. salina*; (B) pH curve; (C) *F_v_/F_m_* curve; (D) β-carotene content. The shaded areas indicate the standard error of the line values.

After 5 days of cultivation with supply of two different carbon sources, the growth showed significant differences in both OD_680_ (*p* < 0.01) and DW (*p* < 0.01). The initial ODs were similar (0.78 for NaHCO_3_-based and 0.79 for CO_2_-based) and then increased to 5.65 ± 0.17 and 6.58 ± 0.23, respectively (Table 6), and the corresponding final DWs were 0.89 ± 0.10 and 1.09 ± 0.08 g/l, respectively (Table 6). The productivity in cultures with NaHCO_3_ and CO_2_ were 0.18 and 0.21 g/l^-1^/d-1, respectively. Clearly, the CO_2_-based mode provided a better growth environment for *D. salina*, resulting in a 14.2% and 22.4% higher OD and DW than the NaHCO_3_-based mode. The pH varied from 6.7 ± 0.1 to 8.3 ± 0.1 in the CO_2_-based mode, while the pH varied from 8.0 ± 0.11 to 9.5± 0.1 in the NaHCO_3_-based mode (Fig. 6B).

Moreover, the 200 mM bicarbonate had positive effects on the productivity of target value chemicals, β-carotene, showing the highest carotenoid concentration in the NaHCO_3_-based condition, 4.7% (41.5 ± 0.2 mg/l)(Fig. 6D, Table 6), this value was significantly higher than that with CO_2_-based condition, 2.2% (23.8 ± 0.3 mg/l). Also, the difference between the DW and β-carotene content in Figs. 6 and 2 should be due to microelement optimization, as evidenced in Fig. 5 and Table 4.

The changing patterns in *F_v_/F_m_* are depicted in Fig. 6C, and the *F_v_/F_m_* of *D. salina* changed periodically following changes in light under two carbon supply conditions, but exhibited similar patterns. Notably, the *F_v_/F_m_* followed ‘sine’ trends during the whole culture light/dark period. As for 2% CO_2_-based condition, during the light period, the *F_v_/F_m_* decreased quickly from 0.73 to the lowest value 0.68 within the first 3 h and then increased gradually to the highest value (0.77) until the darkness period. During the 10 h darkness period, the *F_v_/F_m_* decreased gradually but was significantly higher than that in the 14 h illumination period. For the NaHCO_3_-based culture condition, it was found that lowest *F_v_/F_m_* values were significantly lower than CO_2_-based mode (*p* = 0.0003), and the lowest value of *F_v_/F_m_* in the CO_2_-based mode was 0.05 higher than in the NaHCO_3_-based culture.

## Discussion

Although several studies have indicated that *D. salina* grew well in high concentration of bicarbonate, this study is the first one that reported *D. salina* can accumulate a good amount of β-carotene under such cultivation conditions. Table 7 compared the β-carotene content and β-carotene yield of *Dunaliella* strains in available literature. For *D. salina* CCAP 19/18, the strain used in this study, it accumulated only 2.26 mg/l β-carotene when cultured with 20 mM NaHCO_3_ [20]. Also, the β-carotene yield obtained in this study is much higher than previous studies with other strains in *Dunaliella*. For example, it is about 4-fold of the yield of *D. salina* V-101 with 100 mM NaHCO_3_ [12], and about 1.6-fold of *Dunaliella* sp. with 60 mM NaHCO_3_ [7]. Actually, 200 mM NaHCO_3_ supported better cell growth than other concentrations, but it is lower than that with 2% CO_2_ (Table 6). Since 200 mM bicarbonate resulted in a decreased value of *F_v_/F_m_* (Fig. 6C), it is considered as a stress, but this is favorable for β-carotene production. However, the commonly observed stagnant growth under stress was not found in this study, since the obtained DW of 0.71 ± 0.05 g/l^-1^ is quite comparable with those observed without stress [15]. Thus, this study provided a feasible approach for β-carotene production from *D. salina* with bicarbonate as carbon source.

Precipitation appeared in culture with 200 mM NaHCO_3_ along with 3.0 mM Ca^2+^ and 5.0 mM Mg^2+^, since they react with excessive CO_3_^2-^ at high pH (equilibrium HCO_3_^-^ + OH^-^ → CO_3_^2-^ + H_2_O). Reducing the concentration of Ca^2+^ and Mg^2+^ to 0.3 mM and 0.5 mM, respectively, was proved to be an effective approach to avoid precipitation, and showed no significant reduction of biomass and β-carotene accumulation. In order to reduce production cost, seawater with NaHCO_3_ supply may be used for *D. salina* cultivation, in which Ca^2+^ and Mg^2+^ concentration is about 9-12.5 mM, and 80.5 mM, respectively [21, 22]. These are much higher than 0.3 mM and 0.5 mM, thus excessive Ca^2+^ and Mg^2+^ need to be removed via pretreatment. Precipitation with carbonate may be used as a simple method, and CO_2_ bubbling could regenerate bicarbonate afterwards, which may provide inorganic carbon as in this study [23].

This study first reported the effect of microelements on β-carotene accumulation in *D. salina* when NaHCO_3_ is used as carbon source. The result indicated FeCl_3_·6H_2_O has a negative effect on cell biomass and β-carotene content with bicarbonate as carbon source. These findings were in accordance with previous research with CO_2_ as carbon source, and the amount of FeCl_3_·6H_2_O supplied in this study may induce the generation of active oxygen molecules, and result in a negative effect on biomass and positive effect on β-carotene accumulation [11, 19]. As shown in this study, CoCl_2_·6H_2_O has negative effect on cell growth but positive effect on β-carotene accumulation. There was no report on this topic when *D. salina* is cultivated with CO_2_, but the study on *Platymonas subcordiforus*, *Chaetoceros curvisetus* and *Skeletonema costatum* did show that Co^2+^ inhibition to cell growth in that it affects the interactions among the thylakoid membrane protein-pigment complexes, and obstructs the reaction center of PSII [24]. Also, it was reported that Co^2+^ contributes to the accumulation of carotenoids in *Pavlova viridis*, since it is an oxidative stress-inducing factor to react with hydrogen peroxide through a Fenton-type reaction to generate hydroxyl radicals [25], and these findings are in accordance with this study. This study also indicated that NaVO_3_ concentration had positive effect on biomass but had no significant effect on β-carotene accumulation when cultured with NaHCO_3_, and there was no previous report on this in *D. salina* cultivated with CO_2_. It was reported that 2.5 mM NaVO_3_ promoted astaxanthin production in *H. pluvialis*, and the possible mechanism is the inhibited expression of PTPases (Protein Tyrosine Phosphatases) by NaVO_3_ [26-28]. However, the NaVO_3_ concentration used in this study was only 2.62 μM, which may be too low to induce β-carotene accumulation.

The mechanism of improved β-carotene content under NaHCO_3_-based culture was unknown and there is little information available on the responses of photosynthetic electron flow, especially in photosystem II (PSII) to NaHCO_3_-based in *D. salina*. In the present study, the variation of *F_v_/F_m_* exhibited similar patterns under two carbon supply conditions, but the NaHCO_3_-based culture resulted in lower *F_v_/F_m_* values than that of CO_2_. Also, the pH value was higher at NaHCO_3_-based culture than that of CO_2_. Higher β-carotene accumulation may be attributed to both high concentration NaHCO_3_ and high pH. It was reported that higher extracellular NaHCO_3_ concentration leads to a higher intracellular pH, which may damage or inhibit the enzymes involved in photosynthesis and reduce the efficiency of PSII photosystem (*F_v_/F_m_*) [29]. Also, it was reported that higher NaHCO_3_ concentration (above 0.6 mM) in the culture could inhibit the extracellular carbonic anhydrase (CA) activity, which is an important enzyme catalyzing the reversible dehydration of HCO_3_^-^ to CO_2_, and decline of CA activities has significant inhibition of effective quantum efficiency of PSII, and thus reduce the value of *F_v_/F_m_* [30]. It was reported that decreased PSII activity results in the increase of ROS concentration, and β-carotene is synthesized to scavenge the ROS [31]. This may be the reason why increased β-carotene content was observed in culture with high concentration of NaHCO_3_. However, the connection between ROS and *F_v_/F_m_* under NaHCO_3_ stress in microalgae is still unknown and further in-depth research is needed to disclose the mechanism.

From the above results, FeCl_3_·6H_2_O, NaVO_3_ and CoCl_2_·6H_2_O concentrations significantly influenced *D. salina* biomass production with NaHCO_3_ as carbon source, which was not reported in cultivation with CO_2_. The notable difference in *F_v_/F_m_* value between cultivations with bicarbonate and CO_2_ indicates that NaHCO_3_ acts as a stress factor for β-carotene production more so than CO_2_, and may make it useful in an easy and effective β-carotene induction method.

## Supplemental Materials

Supplementary data for this paper are available on-line only at http://jmb.or.kr.

## Figures and Tables

**Table 1:** Different concentrations of Ca^2+^, Mg^2+^ investigated in this study.

No.	Ca^^2+^^ (mM)	Mg^^2+^^ (mM)
A	0.3	0.5
B	0.6	1.0
C	1.2	2.0
D	1.8	3.0
E	2.4	4.0
F	3.0	5.0

**Table 2:** The coded and real values of the independent variables in the central composite design.

Variables	Unit	-2	-1	0	1	2
FeCl_3_•6H_2_O	μM	1.85	5.92	9.99	14.1	18.1
CoCl_2_•6H_2_O	μM	0.34	0.97	1.60	2.23	2.86
NaVO_3_	μM	0.33	0.9	1.48	2.05	2.62

**Table 3:** The central composite design of the significant (in coded level) with DW and β-carotene yield as responses.

Run	FeCl_3_•6H_2_O	CoCl_2_•6H_2_O	NaVO_3_	DW (g/l)	β-carotene (%)
1	1	1	-1	0.645	4.35
2	1	-1	1	0.637	4.27
3	-1	1	1	0.685	4.76
4	-1	-1	-1	0.664	4.27
5	-2	0	0	0.71	4.28
6	2	0	0	0.629	4.59
7	0	-2	0	0.691	4.16
8	0	2	0	0.665	4.55
9	0	0	-2	0.671	4.21
10	0	0	2	0.708	3.85
11	0	0	0	0.695	3.97
12	0	0	0	0.696	3.94
13	0	0	0	0.707	3.86
14	0	0	0	0.69	4.02
15	0	0	0	0.709	3.86

**Table 4:** The values of coefficients in the second-order polynomial and the associated statistical test for DW and β-carotene.

Variable	DW			β-carotene		
	
	F-value	*p*-value	Estimate	F-value	*p*-value	Estimate
Model	12.06	0.0068	0.7	12.11	0.0067	3.96
A- FeCl_3_•6H_2_O	36.44	0.0018	-0.029	3.05	0.0412	0.1
B- CoCl_2_•6H_2_O	3.75	0.1104	-9.19E-03	5.52	0.0657	0.14
C- NaVO_3_	7.6	0.04	0.013	4.7	0.0824	-0.13
AB	2.15	0.2027	9.83E-03	7.66	0.0395	-0.23
AC	6.01	0.0579	-0.016	3.09E-03	0.9578	-4.61E-03
BC	3.14	0.1366	-0.012	1.348E-07	0.9997	-0.00003048
A^2	27.2	0.0034	-0.018	70.58	0.0004	0.36
B^2	15.77	0.0106	-0.014	15.27	0.0113	0.17
C^2	5.23	0.0709	-7.82E-03	0.004034	0.9518	0.002685

**Table 5:** Comparison of experimental and predicted DW and β-carotene content at optimal culture conditions.

Culture conditions		DW (g•L^-1^)	β-carotene content (%)
Optimal for DW	Predicted	0.86	4.11%
200 mM NaHCO_3_, 0.45 mM MgCl_2_•6H_2_O,0.05 mM MgSO_4_•7H_2_O, 0.3 mM CaCl_2_•2H_2_O, 1.85 µM FeCl_3_, 1.5 µM NaVO_3_, 1.6 µM CoCl_2_ •6H_2_O	Experimental data	0.77±0.01	4.23%±0.01
All other conditions were as described in section Materials and Methods	Deviation (%)	-10.00	+2.90
Optimal for β-carotene content	Predicted	0.76	4.86%
5.92 µM FeCl_3_, 2.0 µM NaVO_3_, 2.2 µM CoCl_2_ •6H_2_O, all other conditions were as described for “optimal DW”	Experimental data	0.69±0.02	4.78%±0.04
	Deviation (%)	-9.20	-1.60
Optimal for DW & β-carotene	Predicted	0.78	4.73%
5.92 µM FeCl_3_, 2.0 µM NaVO_3_, 2.2 µM CoCl_2_ •6H_2_O, all other conditions were as described for “optimal DW”	Experimental data	0.71±0.05	4.52%±0.04
	Deviation (%)	-9.00	-4.60

**Table 6:** Comparison of biomass, β-carotene yield, biomass productivities in cultivation with different carbon sources.

Treatment	Day	OD_680_	Dry weight (g/l)	β-carotene yield (mg/l)	Biomass productivity (g/l/d^-1^)
2% - CO_2_	0	0.78 (0.01)	0.16 (0.02)	1.04 (0.03)	
2% - CO_2_	5	5.65^**^(0.17)	1.09^*^ (0.08)	23.8^**^ (0.5)	0.21
200 mM NaHCO_3_	0	0.79 (0.02)	0.16 (0.02)	1.04 (0.03)	
200 mM NaHCO_3_	5	6.58^**^(0.23)	0.89^*^ (0.10)	41.5^**^ (0.2)	0.18

Values are mean (±SD) of *n* = 3 cultivations per treatment, *represent the significant effect (*p* < 0.05) and ** represent the very significant effect (*p* < 0.01)

**Table 7:** β-carotene accumulation by strains of *Dunaliella* under varied cultivation conditions.

Microalgae	Initial cell density	L/D cycle	Light intensity (µmol•m^-2^•s^-1^)	Culture time (d)	β-carotene content	β-carotene yield (mg/l)	Carbon source	Reference
*D. salina* V-101	-	16/8	50	7	0.05%	8.25±0.01	100 mM NaHCO_3_	Ramachandran Svasanini *et al*,2018 [12]
*D .salina* CCAP 19/30	-	12/12	200	7	-	1.2	10 mM NaHCO_3_	Yanan Xu *et al*,2016
*D. salina* UTEX 2538	-	12/12	1000	5	-	13.2	10 mM NaHCO_3_	Yanan Xu *et al*,2018(Xu *et al*. 2018)
*Dunaliella sp.*	0.1	-	340	17	-	20.43±2.84	60 mM NaHCO_3_	Ga-Yeong Kim *et al*,2017 [7]
*Dunaliella sp.*	0.1	16/8	22	28	0.18%	7.10±0.08	150 mM NaHCO_3_	Srinivasan *et al*,2015 [9]
*D. salina* CCAP 19/18	0.2×10^6^	12/12	200	7	4.50%	32.0	200 mM NaHCO_3_	This study
