# Supplementary material for: β-Carotene Production from Dunaliella salina Cultivated with Bicarbonate as Carbon Source
Source: J Microbiol Biotechnol. 2020 Mar 13;30(6):868–77. doi: 10.4014/jmb.1910.10035 (PMC9728381; doi:10.4014/jmb.1910.10035)
Supplement: Supplementary file 1 [file JMB-30-6-868-supple.pdf]

J. Microbiol. Biotechnol.
https://doi.org/10.4014/jmb.1910.10035

Table S1 Variables to be screened in Plackett-Burman design

	Factor Name	Unit	Low(-)	High(+)
	$FeCl_3·6H_2O$	μM	9.99	19.9
	H_3BO_3	μM	93	185
C	$ZnSO_4·7H_2O$	$mg·L^{-1}$	0.5	1.0
D	$CoCl_2·6H_2O$	$mg·L^{-1}$	0.8	1.6
E	$CuSO_4·5H_2O$	$mg·L^{-1}$	1.0	0.5
F	$MnCl_2·4H_2O$	$mg·L^{-1}$	5.0	10
G	$NaMoO_4·2H_2O$	$mg·L^{-1}$	1.0	2.0
H	$NaVO_3$	$mg·L^{-1}$	0.74	1.48
